# Global research trends and hotspots in aspirin studies (2014–2024): a bibliometric perspective

**DOI:** 10.3389/fphar.2025.1513318

**Published:** 2025-05-16

**Authors:** Ning Sun, Bochao Jia, Rui Wei, Taiwei Lou, Zirong Li, Xiaowei Nie, Xujie Wang, Wenxiao Yu, Qiuyan Li

**Affiliations:** ^1^ Post-doctoral Research Station, Xiyuan Hospital, China Academy of Chinese Medical Sciences, Beijing, China; ^2^ Department of Cardiovascular, Guang’anmen Hospital, China Academy of Chinese Medical Sciences, Beijing, China; ^3^ Department of General Medicine, Beijing University of Chinese Medicine Third Affiliated Hospital, Beijing, China; ^4^ Department of Andrology, Xiyuan Hospital, Beijing, China

**Keywords:** aspirin, cardiovascular disease, bibliometrics, prevention, risk

## Abstract

**Background:**

Aspirin, as one of the most important drugs in medical history, has been continuously explored for over 3,000 years. This study employs bibliometric analysis to examine the research hotspots and trends on aspirin over the past decade.

**Methods and results:**

This study retrieved articles and reviews on aspirin from the Web of Science database, covering the period from 2014 to 2024. R software and CiteSpace were employed for visual analysis, revealing trends in publication volume, collaborations, core journals, and keywords distributions. In the past decade, a total of 19,504 papers authored by 88,600 researchers were published, citing 460,704 references. The U.S., China, and Italy lead in publications, with Canada and Australia showing strong collaboration. The authors with the highest contributions include BHATT DL, STEG PG, and WANG YJ. Research hotspots and trends include the following three points: the development of expert recommendations for the use of aspirin in primary cardiovascular prevention into personalized and shared decision-making between doctors and patients; the ongoing need for more evidence regarding the effects of aspirin on different tumors; and the sustained focus on aspirin-related respiratory diseases in future research.

**Conclusion:**

Aspirin, a classic drug, continues to have a substantial number of publications, underscoring its lasting impact. The United States, China, and Italy play a leading role in this field. However, there is still a long way to go, and research that is more targeted and beneficial for different refined populations may be a future trend.

## Introduction

The history of aspirin dates back more than 3,000 years, when people first discovered that the bark of the willow tree possessed fever-reducing and pain-relieving properties (Antoniadou et al., 2021). In 1763, Edward Stone, an English clergyman, systematically documented these effects (Wood, 2015). The breakthrough in aspirin’s development came in 1897 when German chemist Felix Hoffmann successfully synthesized acetylsalicylic acid, later known as aspirin, marking a significant milestone in pharmaceutical history. Decades later, British pharmacologist John Vane elucidated its mechanism of action, revealing that aspirin exerts its effects by inhibiting the synthesis of prostaglandins. Recognized as one of the three most iconic drugs in modern medical history—alongside penicillin and diazepam—aspirin has played a pivotal role not only in pain management and anti-inflammatory treatment but also in the prevention of cardiovascular and cerebrovascular diseases (Nelson and Black, 2024).

Aspirin has been the subject of extensive medical research, with scientists continually investigating its multifaceted therapeutic potential and associated risks (Werz et al., 2024). Initially studied for its antipyretic and analgesic properties, aspirin’s subsequent discovery as a platelet aggregation inhibitor established its pivotal role in cardiovascular disease prevention. As research progressed, increasing attention has been paid to evaluating the benefit-risk balance of long-term aspirin therapy. Recent investigations have expanded into several promising areas: (1) the potential chemopreventive effects against various malignancies, particularly colorectal cancer; (2) its complex relationship with respiratory conditions, notably aspirin-exacerbated respiratory disease (AERD) (Buchheit et al., 2024; Barinda et al., 2024); and (3) the refinement of clinical applications for different populations. While aspirin’s efficacy in secondary cardiovascular prevention is well-documented, primary prevention strategies require more nuanced, individualized approaches to optimize outcomes while mitigating adverse effects - a crucial direction for future research and clinical practice (Mignozzi et al., 2025; Zheng and Roddick, 2019; Huang et al., 2024; Chipalkatti et al., 2024). Emerging evidence suggests aspirin may exhibit protective effects against colorectal and other gastrointestinal cancers. However, the scientific community emphasizes the need for more rigorous clinical trials to substantiate preliminary findings regarding breast and prostate cancer prevention. Concurrently, aspirin-associated respiratory conditions, including asthma and nasal polyposis, remain important research foci that may present ongoing clinical challenges (Mastalerz et al., 2025; Jermihov et al., 2024). These diverse research avenues underscore aspirin’s complex pharmacological profile and highlight critical gaps requiring further investigation.

Conducting a comprehensive literature review and identifying research hotspots in the extensive field of aspirin studies presents significant challenges, particularly within constrained timeframes. To address this, we employed bibliometric analysis using R software and CiteSpace to systematically evaluate aspirin-related publications from the past decade. This quantitative approach enables efficient visualization of evolving research trends and knowledge structures, providing researchers with valuable insights into the most active areas of investigation and emerging frontiers in aspirin research.

## Materials and methods

### Dataset establishment

**FIGURE 1:**
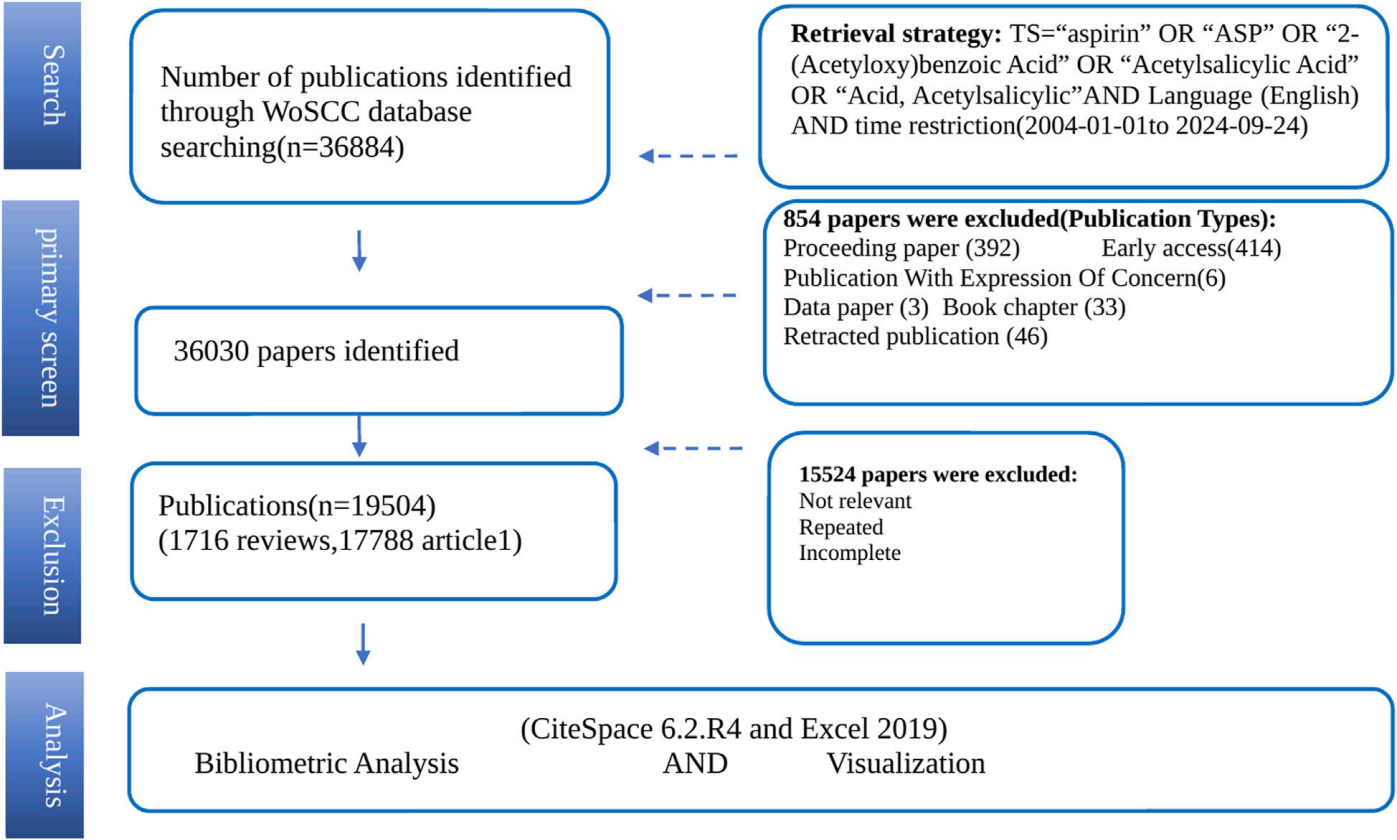
Study flow chart. TS, indicates topic search; and WoSCC, web of science core collection.

The WoSCC (Web of Science Core Collection) database is recognized as one of the most comprehensive, systematic, and authoritative repositories in the world, frequently used for visualizing scientific literature. To obtain data that is both representative and accurate, the retrieval parameters were defined as follows: TS = “aspirin” OR “ASP” OR “ASA” OR “2-(Acetyloxy) benzoic Acid” OR “Acetylsalicylic Acid” OR “Acid, Acetylsalicylic.” For subsequent analysis, the citation edition was restricted to the Science Citation Index Expanded (SCI-Expanded), and only articles and reviews published in English were included. All bibliographic records, including titles, authors, keywords, institutions, abstracts, references, and publication years, were archived in plain text format. Data retrieval was conducted on 24 September 2024. Two authors (SN and JBC) independently carried out the data identification and screening of the retrieved literature. In cases of disagreement, a third author (YWX) was consulted for arbitration. The data screening process is shown in Figure 1. Since the research data was sourced from an open database, ethical considerations were not applicable.

### Database analysis

The screened literature data was imported into Biblioshiny and CiteSpace (version 6.2.R4) for visual analysis. Biblioshiny, powered by the R software 4.3.2 and the Bibliometrix package (version 4.1.4), provided an intuitive and well-structured interface for quantitatively analyzing annual publications. It also facilitated the visualization of national collaborations, core journals, author publication timelines, and thematic maps of keywords within the realm of aspirin research. CiteSpace specializes in the co-occurrence network visualization of scientific knowledge. In this study, CiteSpace was used for co-occurrence maps, burst map, and keyword clustering of keywords. The selection criteria we chose included the G-index, with a K value set to 3. For the keyword clustering algorithm, we chose the Latent Semantic Index (LSI).

## Results

### Publication and citation summary

**FIGURE 2:**
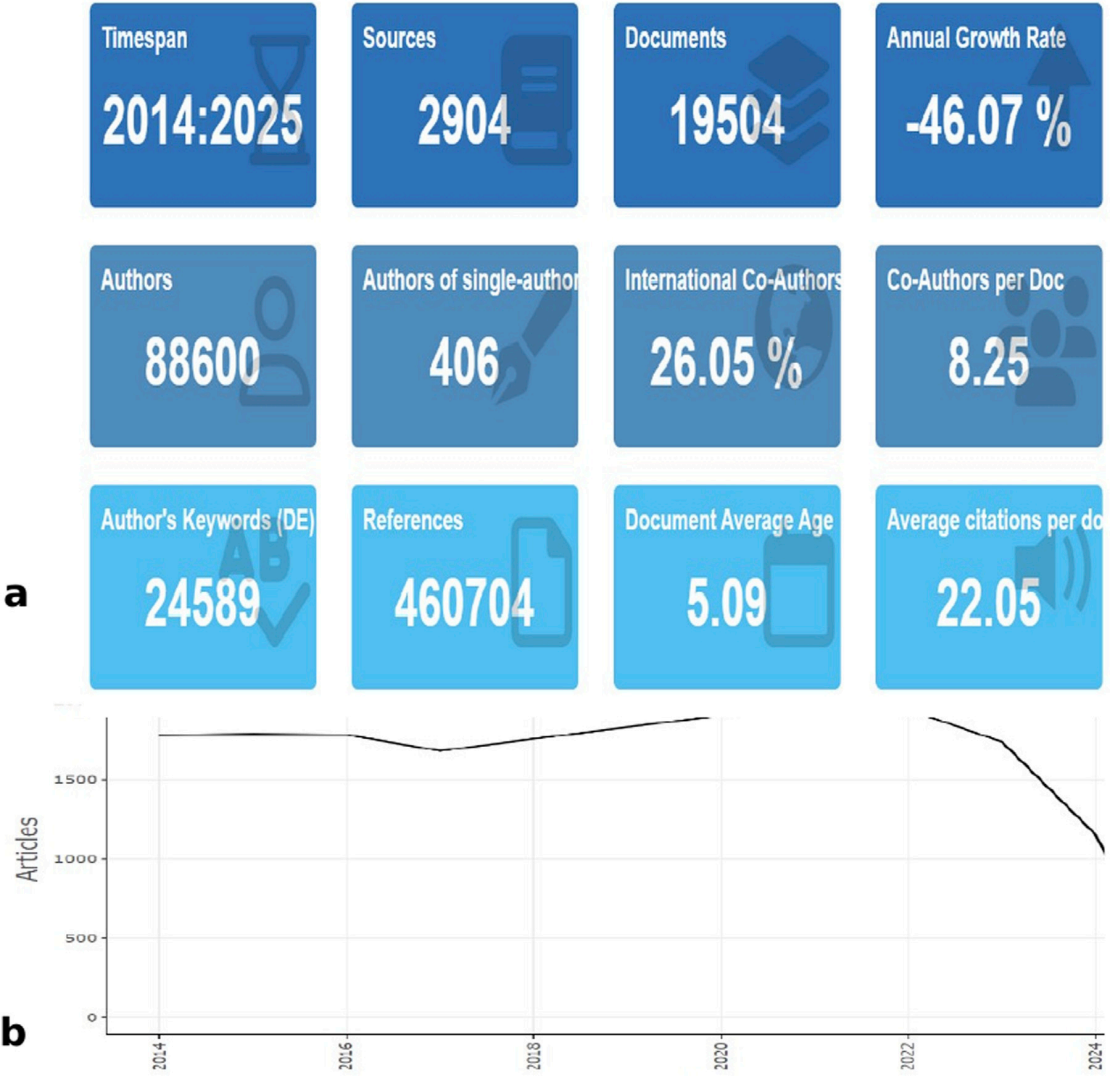
Analysis of annual publications. **(a)** Main information. **(b)** Analysis of annual scientific production related to aspirin.

Figure 2a illustrates that 88,600 authors published a total of 19,504 papers related to aspirin between 1 January 2014, and 1 September 2024, which includes 17,788 articles and 1,716 reviews. Over this period, a total of 24,589 keywords were involved, 460,704 references were cited, and the average citation frequency of the literature was 22.05 times. The annual publication volume within distinct time frames serves as a quantitative indicator of the developmental trajectory within a research domain. Based on this, the evolution of the aspirin field can be segmented into three discernible stages (Figure 2b). The number of aspirin-related publications exhibited a generally stable upward trend from 2014 to 2020. However, starting in 2022, the publication volume for aspirin showed a significant decline, although it still maintained over 1,600 articles per year.

### Analysis of countries or regions

**FIGURE 3:**
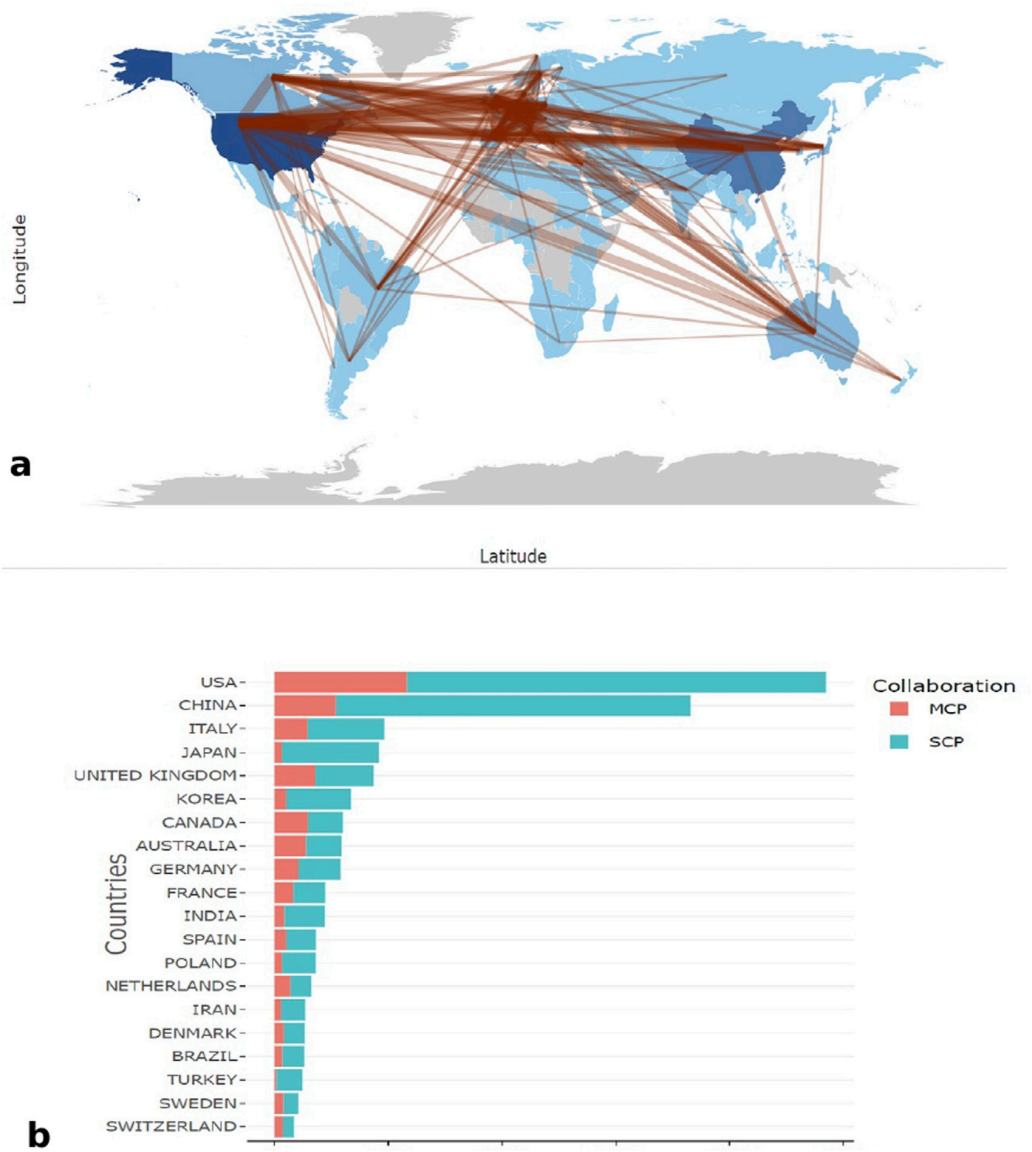
Disrtibution map of countries. **(a)** The country collabration map. **(b)** The proportion of SCP and MCP in the top ten countries ranked by publication output. SCP: single country publication. MCP: multiple country publication.

The top three countries with the highest number of publications were the United States, China, and Italy. The United States published the most papers (4,850), followed by China (3,656) and Italy (965) (Figure 3; Table 1). Figure 3a presents a collaboration map among countries, where the thickness of the lines represents the degree of collaboration between them. Thicker lines indicate more frequent collaborations, while thinner lines indicate lower collaboration frequencies. Figure 3b displays the number of Single Country Publications (SCP) and Multiple Country Publications (MCP) of top 10 countries or regions. Supplementary Table S1 shows that the centrality values for Germany, France, and the Netherlands exceed 0.1, indicating that these three nodes have stronger connections with other nodes in the network.

**TABLE 1:** Top 10 contributing countries/regions related to aspirin.

Country	Articles	Articles %	SCP	MCP	MCP %
USA	4850	24.9	3687	1163	24
China	3656	18.7	3117	539	14.7
Italy	965	4.9	676	289	29.9
Japan	918	4.7	852	66	7.2
United Kingdom	868	4.5	511	357	41.1
Korea	675	3.5	575	100	14.8
Canada	602	3.1	309	293	48.7
Australia	586	3	312	274	46.8
Germany	575	2.9	368	207	36
France	448	2.3	284	164	36.6

### Analysis of authors

**FIGURE 4:**
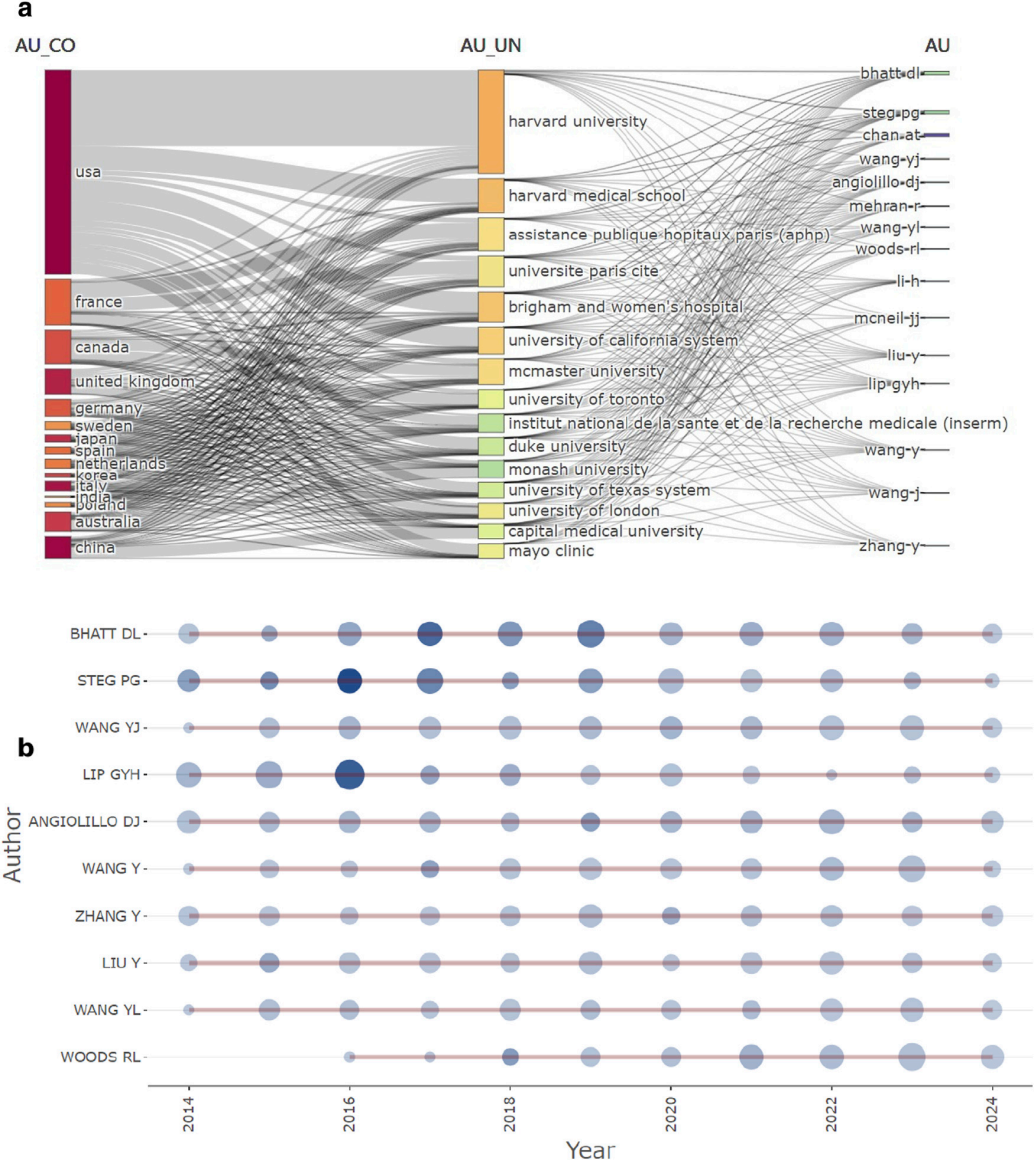
Disrtibution map of authors. **(a)** Three-field plot-country, institution, authors. **(b)** Author’s production over time.

Based on publication volume and citation counts, we ranked the authors and compiled Table 2, which showcases the top ten authors. The author with the highest number of publications is BHATT DL (n = 163), followed by STEG PG (n = 142) and WANG YJ (n = 142). In terms of citation frequency, STEG PG ranked first with 4,236 citations, followed by BHATT DL with 3,800 citations and STOREY RF with 2,164 citations (Table 2). Figure 4A illustrates the relationships among authors, countries, and institutions. Figure 4b presents the timeline of authors’ publications. The larger the circle, the greater the number of aspirin-related papers published by the author; conversely, the darker the circle’s color, the higher the citation frequency. We can observe that STEG PG, ANGIOLILLO DJ, and LIP GYH achieved a high number of publications and citation counts as early as 2014, establishing themselves as pioneers in the field during that time. From 2014 to 2010, BHATT DL and LIP GYH consistently published aspirin-related works. Between 2016 and 2017, both reached their peak citation counts. Notably, WOODS RL began publishing articles related to aspirin only in 2016, but their publication volume has been steadily increasing, allowing them to emerge as a rising star in this field.

**TABLE 2:** The most influential authors in the field of aspirin.

Authors	Articles	Articles Fractionalized	Author	Citations
Bhatt DL	163	15.10	Steg PG	4236
Steg PG	142	9.66	Bhatt DL	3800
Wang YJ	142	14.20	Storey RF	2164
Lip GYH	139	22.13	Valgimigli M	2086
Angiolillo DJ	133	18.31	Mehran R	1984
Wang Y	121	16.67	Vranckx P	1969
Zhang Y	118	15.44	Angiolillo DJ	1788
Liu Y	115	14.78	Mauri L	1698
Wang YL	107	10.10	Windecker S	1663
Woods RL	106	8.26	Parkhomenko A	1632

### Analysis of journals and most cited papers

**FIGURE 5:**
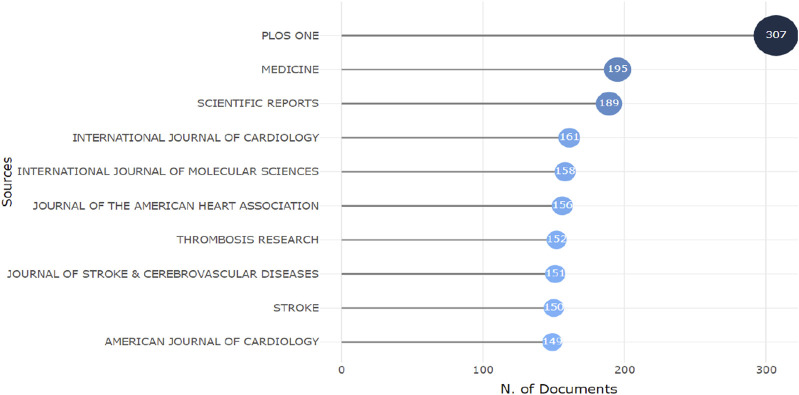
Top 10 Journal most publication related to aspirin.

**FIGURE 6:**
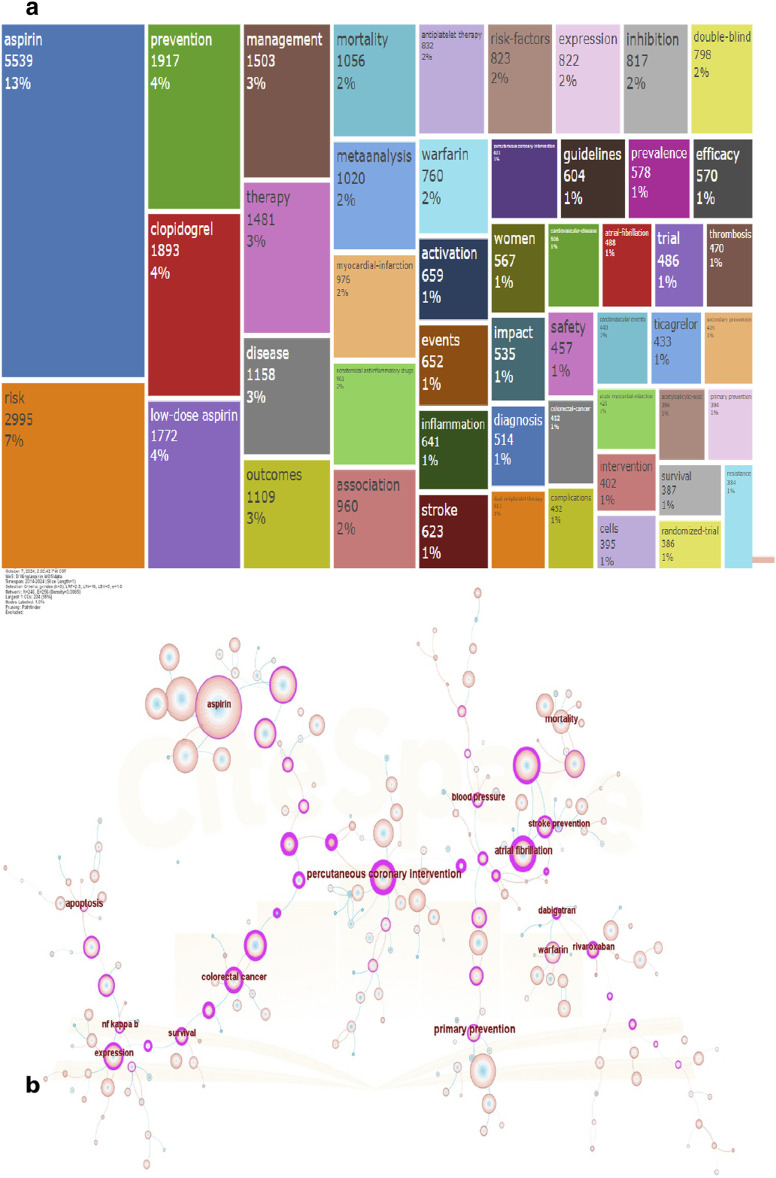
Visualization of keywords. **(a)** Frequency and percentage of keywords. **(b)** Co-occurrence visualization of keywords.

A total of 2,904 journals published academic papers on aspirin. Figure 5 shows the top ten journals with the highest publication volume. The leading journal is PLOS ONE, which published 307 papers on aspirin, followed by Medicine with 195 papers and Scientific Reports with 189 papers. PLOS ONE is a multidisciplinary journal that encompasses natural sciences, medical research, engineering, as well as related social sciences and humanities. It encourages interdisciplinary research to strengthen the foundations of science. The journal Medicine covers a wide range of medical disciplines, including anesthesiology, neurology, cardiovascular health, nutrition, and public health. Scientific Reports primarily focuses on original research in the fields of natural sciences and clinical sciences (Table 3). The most cited article is titled “2019 ACC/AHA Guideline on the Primary Prevention of Cardiovascular Disease: Executive Summary: A Report of the American College of Cardiology/American Heart Association Task Force on Clinical Practice Guidelines,” authored by Donna K. Arnett and published in the journal Circulation in 2019. This article is authored by several authoritative experts in the field of cardiovascular disease and is based on multiple high-level pieces of evidence to establish guidelines for the primary prevention of cardiovascular disease. It covers recommendations for preventing cardiovascular disease in different populations, addressing various aspects such as diet, exercise, and pharmacological treatment, thereby providing guidance for clinicians. The second most cited article is “2019 ACC/AHA Guideline on the Primary Prevention of Cardiovascular Disease: A Report of the American College of Cardiology/American Heart Association Task Force on Clinical Practice Guidelines,” followed closely by “2015 ESC Guidelines for the Management of Acute Coronary Syndromes in Patients Presenting Without Persistent ST-Segment Elevation.” After merging synonymous keywords, such as combining “ASP” with “aspirin,” a total of 24,589 keywords were extracted from 19,504 papers. As shown in Figure 6a, the most common molecular keywords were aspirin (N = 5,539, 13%), risk (N = 2,995, 7%), prevention (N = 1,917, 4%), clopidogrel (N = 1,893, 4%), low-dose aspirin (N = 1,772, 4%), management (N = 1,503, 3%), and therapy (N = 1,481, 3%). The most frequent keywords related to pathological processes were growth (N = 1,127), metastasis (N = 947), and progression (N = 733). Meanwhile, breast cancer (N = 1,144), colorectal cancer (N = 682), and pancreatic cancer (N = 502) were the diseases most closely associated with cancer-associated fibroblasts (CAFs). Figure 6b is a visualization of keyword co-occurrence. The larger the node, the higher the frequency of that keyword’s occurrence. The thicker the line connecting two nodes, the stronger the relationship between them. The colors of the nodes and lines represent the time of keyword occurrence; colors closer to pink indicate more recent appearances, while colors nearer to blue signify earlier occurrences.

**TABLE 3:** The top 10 articles related to aspirin with the highest number of citations.

Paper	DOI	Total Citations	TC per Year	Normalized TC
Arnett DK, 2019, Circulation	10.1161/CIR.0000000000000677	4635	772.50	155.38
Arnett DK, 2019, Circulation-a	10.1161/CIR.0000000000000678	4635	772.50	155.38
Roffi M, 2016, Eur Heart J	10.1093/eurheartj/ehv320	3883	431.44	105.67
Piepoli MF, 2016, Eur Heart J	10.1093/eurheartj/ehw106	3077	341.89	83.74
Piepoli MF, 2016, Atherosclerosis	10.1016/j.atherosclerosis.2016.05.037	2443	271.44	66.48
Malfertheiner P, 2017, Gut	10.1136/gutjnl-2016-312288	1884	235.50	56.26
Levine GN, 2016, J Am Coll Cardiol	10.1016/j.jacc.2016.03.513	1520	168.89	41.37
Eikelboom JW, 2017, New Engl J Med	10.1056/NEJMoa1709118	1518	189.75	45.33
Mauri L, 2014, New Engl J Med	10.1056/NEJMoa1409312	1491	135.55	44.66
Bonaca MP, 2015, New Engl J Med	10.1056/NEJMoa1500857	1446	144.60	43.68
Fanouriakis A, 2019, Ann Rheum Dis	10.1136/annrheumdis-2019-215089	1193	198.83	39.99
Valgimigli M, 2018, Eur Heart J	10.1093/eurheartj/ehx419	1119	159.86	40.48
Xi Y, 2021, Transl Oncol	10.1016/j.tranon.2021.101174	1058	264.50	79.79
Chey WD, 2017, Am J Gastroenterol	10.1038/ajg.2016.563	979	122.38	29.23
Cannon CP, 2017, New Engl J Med	10.1056/NEJMoa1708454	967	120.88	28.87
Mol BJ, 2016, Lancet	10.1016/S0140-6736(15)00070-7	922	102.44	25.09
Bäck M, 2019, Nat Rev Cardiol	10.1038/s41569-019-0169-2	910	151.67	30.51
Hankey GJ, 2017, Lancet	10.1016/S0140-6736(16)30962-X	879	109.88	26.25
Crusz SM, 2015, Nat Rev Clin Oncol	10.1038/nrclinonc.2015.105	863	86.30	26.07
Kattoor AJ, 2017, Curr Atheroscler Rep	10.1007/s11883-017-0678-6	838	104.75	25.02
Serhan CN, 2018, J Clin Invest	10.1172/JCI97943	819	117.00	29.62
Costa F, 2017, Lancet	10.1016/S0140-6736(17)30397-5	806	100.75	24.07
Johnston SC, 2018, New Engl J Med	10.1056/NEJMoa1800410	770	110.00	27.85
Chmiela S, 2017, Sci Adv	10.1126/sciadv.1603015	761	95.13	22.72
Xiao Y, 2021, Pharmacol Therapeut	10.1016/j.pharmthera.2020.107753	759	189.75	57.24

**FIGURE 7:**
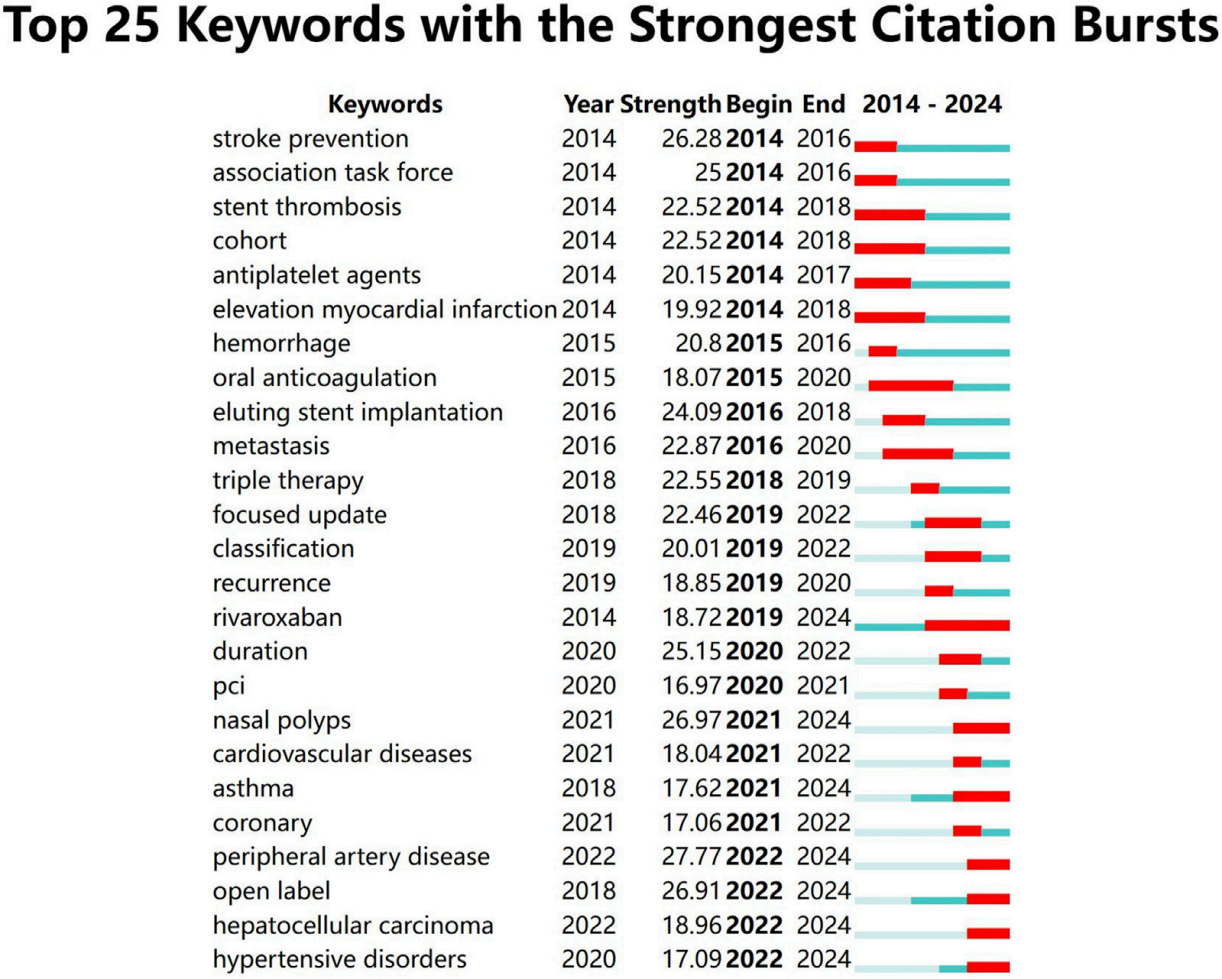
Keywords with the strongest citation bursts.

In the keywords with the strongest citation bursts (Figure 7), The blue line denotes the timeline, 红色 this refers to the sudden increase in citations of specific articles within a certain period. This serves to identify the latest high-profile research keywords in the field of aspirin research. All 25 keywords exhibited strength values significantly greater than 2.0, demonstrating statistically significant prominence during this time period.”

**FIGURE 8:**
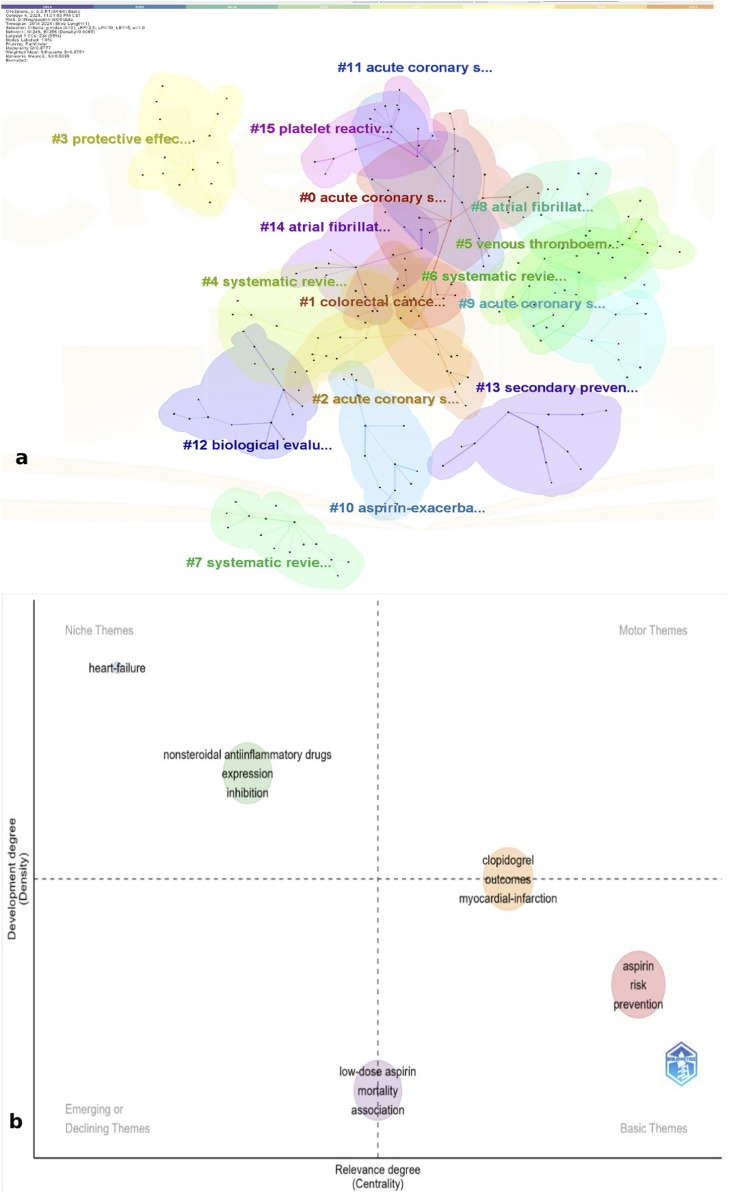
**(a)** Clusters of keywords related to aspirin. **(b)** Thematic map of keywords related to aspirin.

We can observe the evolution of the 25 highlighted keywords over the past decade, starting with a focus on aspirin’s role in the prevention of stroke and thrombosis after coronary stenting. In recent years, there has been a shift toward interest in aspirin-related asthma, nasal polyps, peripheral artery disease, and hepatocellular carcinoma. Figure 8a displays the clustering results of 16 key clusters (cluster 0# to 15#), as indicated by the data in the upper left corner of the figure. The Modularity Q of this clustering is 0.887, and the weighted mean silhouette S is 0.9751. A Modularity Q greater than 0.3 indicates that the community structure is stable, while a silhouette S value greater than 0.5 suggests that the clustering is reasonable. A silhouette S value above 0.7 indicates that the clustering is both meaningful and convincing. The top five clusters are as follows:

Cluster 0 is colored in red, with the main keywords focusing on the primary clinical applications of aspirin and other drugs in the same category. The key terms include percutaneous coronary intervention, acute coronary syndrome, dual antiplatelet therapy, and prasugrel.

Cluster 1 is colored in orange and showcases research on aspirin in the context of cancer. The main keywords include colorectal cancer, breast cancer, chemoprevention, and survival.

Cluster 2 is colored in deep yellow and focuses on the risks associated with aspirin and meta-analyses. The keywords include risk, clopidogrel, therapy, meta-analysis, and disease.

Cluster 3 is colored in light yellow and focuses on the experimental research aspects of aspirin. The keywords include oxidative stress, nitric oxide, apoptosis, *in vitro*, and cells.

Cluster 4 is colored in light green and includes keywords related to preeclampsia. The main terms are preeclampsia, pregnancy, hypertension, and fetal growth restriction. Figure 8b analyzes the thematic map, and the following insights were gleaned:

The themes of outcomes, myocardial infarction, and clopidogrel occupy the first quadrant, indicating their high centrality and prominent status within the research niche.

Themes situated in quadrant four represent potential research hotspots that may become future trends. These themes highlight the risk factors associated with aspirin in disease prevention.

Located in the second quadrant, these themes represent a niche topic characterized by strong professionalism and growing attention. They focus on the inhibitory effects of aspirin on disease occurrence and the genes and protein expressions that mediate the drug’s mechanisms of action.

The themes of low-dose aspirin, association and mortality are situated in the third quadrant, which refers to themes that are either newly developed or are soon to decline.

## Discussion

The analysis of aspirin research from 1 January 2014, to 1 September 2024, reveals important trends in publication and citation patterns. A total of 19,504 papers authored by 88,600 researchers reflected a sustained interest in aspirin’s role in cardiovascular prevention and emerging therapeutic applications. The United States, China, and Italy lead in publication output, while a decline in volume post-2022 suggests possible shifts in research focus. Canada and Australia demonstrate a higher tendency for collaborative research, enhancing the impact of their contributions. Key authors such as BHATT DL and STEG PG have significantly influenced the literature, shaping clinical practices and guidelines. Journals like PLOS ONE play a critical role in disseminating multidisciplinary research, highlighting the importance of collaboration. The most cited articles, particularly those on cardiovascular disease guidelines, emphasize the practical implications of research findings. Keywords analysis indicates a shift from stroke prevention to broader interests in aspirin’s effects on asthma, nasal polyps, and specific cancers, reflecting an expanding research scope. This shift underscores the growing recognition of aspirin’s multifaceted roles, especially in oncology.

### Aspirin for Primary Prevention of Cardiovascular Disease: From a Pillar of Prevention to Individualized Treatment and Shared Decision-Making

Aspirin remains one of the most widely used medications globally. While there is strong evidence supporting its efficacy in the secondary prevention of ischemic events for patients with established cardiovascular disease, its role in primary prevention has been a topic of ongoing debate over the past decade. More than 80 million people worldwide are taking aspirin for the primary prevention of cardiovascular disease (CVD) (Yoo et al., 2024). Although early trials showed the benefits of aspirin in reducing cardiovascular events, more recent studies have challenged these findings, with some even indicating a net harm. As a result, recommendations for aspirin use in primary prevention have undergone significant changes in recent years. In 2002, the U.S. Preventive Services Task Force (USPSTF) recommended that adults with a 5-year coronary heart disease (CHD) risk of 3% or higher should consider taking aspirin. By 2015, the recommendation shifted to suggest that diabetic patients with a 10-year cardiovascular event risk of 5%–10% could consider aspirin use (Class IIB). By 2022, the USPSTF guidelines further evolved, advising that adults aged 40 to 59 with a 10-year cardiovascular disease (CVD) risk greater than 10% make an individualized decision on aspirin use, while for those aged 60 and older, aspirin is not recommended for the primary prevention of CVD (as detailed in Table 4).

**TABLE 4:** The evolution of guidelines or consensus on the use of aspirin for primary prevention of cardiovascular disease.

Year	Guideline development organizations	Recommended content
2002	USPSTF	Consider aspirin in adults with a 5-year risk of coronary heart disease >3%
2002	AHA	Adults with a 10-year cardiovascular risk greater than 3% should consider aspirin
2007	ESC	Adults with an elevated 10-year cardiovascular event risk (SCORE risk score >10%) and controlled blood pressure should consider aspirin
2009	USPSTF	For diabetic patients with a 10-year cardiovascular event risk of 10% and a low risk of bleeding, aspirin is recommended (Recommendation Level: IIA). For diabetic patients with a 10-year cardiovascular event risk of 5% to 10%, aspirin may be considered (Recommendation Level: IIB)
2015	AHA/ADA	Diabetic patients with a 10-year risk of 10%cardiovascular events and a low risk of bleeding · Aspirin recommended (recommended level: IIa) May be considered in patients with diabetes who have a 10-year risk of cardiovascular events between 5% and 10%. Aspirin (recommended grade: IIb)
2016	USPSTF	For patients aged 50 to 90 with a 10-year cardiovascular event risk of ≥10% and a low risk of bleeding, aspirin is recommended (Grade: B)
2016	ESC	Aspirin is not recommended for individuals without evident cardiovascular disease
2019	AHA/ACC	Aspirin is not recommended for adults over the age of 70; for adults aged 40 to 70, aspirin may be considered (Recommendation Level: IIb)
2020	ADA	For diabetic patients aged 50 to 70 with elevated ASCVD risk, aspirin (75-163 mg/d) may be considered after a thorough assessment of the risk/benefit ratio. Aspirin is not recommended for low ASCVD risk populations (including patients under 50 years old) and individuals over 70 years old for primary prevention. N/AA
2022	USPSTF	For adults aged 40 to 59 with a 10-year cardiovascular disease risk of ≥10%, the decision to use low-dose aspirin for primary prevention of cardiovascular disease should be individualized; it is not recommended for adults aged 60 and older to start using low-dose aspirin for cardiovascular primary prevention (Grade: D)

This brings us to the three landmark trials conducted in 2018, which highlighted the potential risks associated with aspirin use in certain populations. These studies suggested that in middle-risk elderly individuals, healthy older adults, and patients with diabetes, the use of aspirin for the primary prevention of cardiovascular disease might increase the risk of bleeding, outweighing the benefits of preventing ischemic heart disease. The ARRIVE trial (Gaziano et al., 2018) was a randomized controlled study conducted across seven countries, involving 12,546 participants. The study included men aged 55 and older, and women aged 60 and older, who were classified as having moderate cardiovascular risk. Participants were given 100 mg of aspirin daily and followed for 5 years. Compared to the placebo group, there was no statistically significant difference in cardiovascular endpoints in the aspirin group. However, the aspirin group had an increased risk of gastrointestinal bleeding. The ASCEND trial (Bowman et al., 2018) enrolled 15,480 patients with diabetes aged 40 and older, who took 100 mg of aspirin daily. Over a median follow-up of 7.4 years, the results showed a 12% reduction in vascular events in the aspirin group. However, this benefit was accompanied by a 29% increase in the risk of major bleeding. The ASPREE trial enrolled 19,114 healthy older adults with a median age of 74 years. The results indicated that taking 100 mg of aspirin daily did not reduce the incidence of cardiovascular events in this population. In fact, it was associated with an increased risk of bleeding and mortality (Hawk and Maresso, 2021).

In addition, the study also showed that the benefits of aspirin may be influenced by multiple factors, including gender, race, and income level. The study by Yoo et al. (2024) indicated that in high-income countries, the risks associated with aspirin use for primary prevention are relatively low, whereas in low-income countries, the bleeding risk is significantly higher (Argenziano et al., 2024). A study focused on the U.S. population found that the benefits of aspirin for primary prevention of cardiovascular disease are associated with gender and race. Specifically, it indicated that only men and Black individuals derive significant benefits from aspirin in this context (Huang et al., 2024).

In the subsequent 2019 guidelines, recommendations for aspirin use in the primary prevention of cardiovascular disease began to shift gradually. Aspirin transitioned from being viewed as a universal preventive medication to a more nuanced approach that emphasizes population-specific strategies, individualized treatment plans, and shared decision-making between healthcare providers and patients. On the basis of shared decision-making between healthcare providers and patients, selecting the appropriate patients and initiating aspirin therapy at the right time is crucial for the prevention of cardiovascular disease (CVD). Overall, patients who are at high risk for CVD but have a low risk of bleeding are more likely to benefit from aspirin use in primary prevention.

#### Aspirin’s dual role in cancer prevention: benefits, mechanisms, and innovations

Long-term use of aspirin for more than 5 years has been shown to reduce the risk of certain cancers, but it may also increase the risk of a few others. In a cohort study spanning 20 years with a total of 1,909,531 participants, long-term aspirin use (≥5 or 10 years) was associated with a risk reduction of over 10% for cancers such as gastrointestinal tumors, pancreatic cancer, small intestine cancer, brain tumors, and thyroid cancer. However, the risk of lung and bladder cancer was found to increase with prolonged aspirin use (Skriver et al., 2024). Among these, the evidence for the protective effects of aspirin against hepatocellular carcinoma and colorectal cancer is more compelling (Chen S. et al., 2024),However, the protective effects of aspirin on other types of tumors still require further in-depth research to be fully understood. For example, the protective effect of aspirin against breast cancer still requires more convincing evidence and further high-quality randomized controlled trials (RCTs) to support this claim. Some studies have suggested that only patients with estrogen receptor-positive (ER-positive) breast cancer may benefit from aspirin use (Chen and Holmes, 2017; Baker and Kartsonaki, 2024; Cairat et al., 2021; Ademi et al., 2013).The mechanisms by which aspirin may prevent tumors could involve multiple factors, including energy metabolism, inflammation, inhibition of platelet aggregation, immune evasion, cellular abnormalities, glycolysis, and cellular programming. Potential targets implicated in these processes may include MCM6, RRM2, TIGIT, PI3K and ARFIP2 (Zheng et al., 2021; Nounu et al., 2021; Tougeron et al., 2014; Liu et al., 2024).

Recent studies have also highlighted the potential of using aspirin in combination with other types of medications for cancer prevention and treatment. For example, combining aspirin with 5-fluorouracil (5-FU) or glutaminase inhibitors has shown promise in enhancing therapeutic outcomes. Cellular metabolic reprogramming has been identified as a crucial mechanism of action for aspirin. This includes the regulation of key metabolic drivers, modulation of enzymes involved in glycolysis and glutamine catabolism, and alterations in nutrient utilization following aspirin exposure. Importantly, since aspirin treatment exposes metabolic vulnerabilities in tumor cells, there is an opportunity to combine aspirin with specific metabolic inhibitors, such as glutaminase inhibitors (Hoskin et al., 2023). Based on the enhanced synergistic antitumor activity and the relationship between inflammation and carcinogenesis, the authors designed chitosan nanoparticles for the co-delivery of 5-fluorouracil (5-Fu), an anticancer agent, and aspirin, a non-steroidal anti-inflammatory drug. This combination aims to induce synergistic antitumor activity by modulating the nuclear factor kappa B (NF-κB)/cyclooxygenase-2 (COX-2) signaling pathway. The results indicated that non-cytotoxic concentrations of aspirin sensitized hepatocellular carcinoma cells to 5-Fu *in vitro*. Specifically, aspirin was found to inhibit NF-κB activation, which in turn suppressed COX-2 expression and prostaglandin E2 (PGE2) synthesis. Moreover, the findings clearly demonstrate that the chitosan nanoparticles loaded with 5-Fu and aspirin enhanced the intracellular concentration of the drugs, leading to synergistic growth inhibition and apoptosis induction in hepatocellular carcinoma cells through the inhibition of NF-κB activation and COX-2 expression (Wang et al., 2020).

#### Aspirin-exacerbated respiratory disease: a persistent clinical challenge ahead

Aspirin-exacerbated respiratory disease (AERD) has been classified as a distinct syndrome. It encompasses a combination of asthma, chronic rhinosinusitis (CRS) with nasal polyps, and acute upper and lower respiratory reactions that occur following the ingestion of aspirin (acetylsalicylic acid, ASA) and other cyclooxygenase-1 (COX-1) inhibitory non-steroidal anti-inflammatory drugs (NSAIDs). AERD is a serious condition characterized by dysregulation of type 2 inflammation (Abud and White, 2024). AERD is characterized by low awareness and high diagnostic delays. In initial consultations, 27% of patients were unaware that they had non-exacerbated respiratory disease (N-ERD), with a median delay in diagnosis of 3 years from the time of awareness (Dages et al., 2023). In the AERD population, elevated levels of innate and IL-6-related cytokines have been detected in the respiratory tract. Both oncostatin M (OSM) and IL-6 are locally produced in nasal polyps and may contribute to pathology by negatively impacting epithelial barrier function. Although IL-4Rα blockers appear to target type 2 inflammation, they also reduce mediators of innate inflammation and epithelial dysregulation, which may enhance the therapeutic efficacy of dupilumab in AERD (Chen C. C. et al., 2024). This highlights the significance of aspirin challenge testing for patients with respiratory diseases exacerbated by non-steroidal anti-inflammatory drugs (NSAIDs) (Förster-Ruhrmann and Olze, 2024). Addictionally, aspirin desensitization and aspirin desensitization therapy (ATAD) are considered classic treatments for this condition (Zhao et al., 2024).

## Limitations

First, the study only searched the Web of Science (WOS) database. While it is widely recognized as an authoritative database globally, this approach may not encompass all relevant studies, leading to certain limitations in comprehensiveness. Second, the data retrieved was limited to information available up until September 2024, meaning that any studies published in October 2024 and beyond are not included in this analysis. Third, our data screening process involved manual selection, which inevitably introduces the potential for subjective bias.

## Conclusion

As one of the most classic drugs, aspirin holds significant research value in medical science. According to the visual analysis conducted using CiteSpace and R software, research on aspirin continues to maintain a substantial volume, even though more than 3,000 years have passed since its discovery, highlighting its significant impact. United States, China and Italy are the leading countries in terms of research; Several well-known and convincing articles in the field of cardiovascular have emerged. Research on aspirin is far from being perfectly concluded. Future efforts are likely to focus on personalized and precision-based approaches.
