# Supplementary material for: Global research trends and hotspots in aspirin studies (2014–2024): a bibliometric perspective
Source: Front Pharmacol. 2025 May 16;16:1513318. doi: 10.3389/fphar.2025.1513318 (PMC12123089; doi:10.3389/fphar.2025.1513318)
Supplement: Supplementary file 1 [file Table1.docx]

**S1. Count of publications of countries**

| **Rank** | **Count** | **Centrality** | **Year** | **Country** |
| --- | --- | --- | --- | --- |
| 1 | 6342 | 0 | 2014 | USA |
| 2 | 3674 | 0 | 2014 | PEOPLES R CHINA |
| 3 | 1722 | 0 | 2014 | ENGLAND |
| 4 | 1509 | 0 | 2014 | ITALY |
| 5 | 1220 | 0.1 | 2014 | GERMANY |
| 6 | 1140 | 0 | 2014 | JAPAN |
| 7 | 1139 | 0.02 | 2014 | CANADA |
| 8 | 907 | 0 | 2014 | AUSTRALIA |
| 9 | 876 | 0.12 | 2014 | FRANCE |
| 10 | 800 | 0 | 2014 | SOUTH KOREA |
| 11 | 731 | 0.14 | 2014 | NETHERLANDS |
| 12 | 723 | 0 | 2014 | SPAIN |
| 13 | 602 | 0 | 2014 | INDIA |
| 14 | 556 | 0 | 2014 | POLAND |
| 15 | 524 | 0 | 2014 | SWEDEN |
| 16 | 466 | 0.05 | 2014 | DENMARK |
| 17 | 462 | 0.02 | 2014 | SWITZERLAND |
| 18 | 413 | 0 | 2014 | BRAZIL |
| 19 | 401 | 0 | 2014 | TAIWAN |
| 20 | 323 | 0.02 | 2014 | AUSTRIA |
| 21 | 305 | 0 | 2014 | SCOTLAND |
| 22 | 301 | 0 | 2014 | IRAN |
| 23 | 298 | 0 | 2014 | TURKEY |
| 24 | 296 | 0.25 | 2014 | BELGIUM |
| 25 | 272 | 0.07 | 2014 | GREECE |
| 26 | 268 | 0 | 2014 | ISRAEL |
| 27 | 239 | 0.1 | 2014 | IRELAND |
| 28 | 222 | 0.02 | 2014 | SAUDI ARABIA |
| 29 | 217 | 0.05 | 2014 | EGYPT |
| 30 | 188 | 0 | 2014 | PAKISTAN |
| 31 | 162 | 0 | 2014 | NORWAY |
| 32 | 158 | 0.14 | 2014 | NEW ZEALAND |
| 33 | 149 | 0 | 2014 | RUSSIA |
| 34 | 145 | 0.05 | 2014 | CZECH REPUBLIC |
| 35 | 136 | 0.13 | 2014 | SOUTH AFRICA |
| 36 | 136 | 0.02 | 2014 | FINLAND |
| 37 | 133 | 0 | 2014 | HUNGARY |
| 38 | 131 | 0.02 | 2014 | ARGENTINA |
| 39 | 127 | 0.24 | 2014 | ROMANIA |
| 40 | 126 | 0.02 | 2014 | PORTUGAL |
| 41 | 126 | 0 | 2014 | SINGAPORE |
| 42 | 118 | 0.02 | 2014 | MALAYSIA |
| 43 | 113 | 0.05 | 2014 | THAILAND |
| 44 | 100 | 0.02 | 2014 | SERBIA |
| 45 | 90 | 0 | 2014 | MEXICO |
| 46 | 79 | 0.5 | 2014 | COLOMBIA |
| 47 | 75 | 0.02 | 2014 | CHILE |
| 48 | 74 | 0 | 2014 | WALES |
| 49 | 68 | 0.02 | 2014 | NORTH IRELAND |
| 50 | 50 | 0.02 | 2014 | CROATIA |
| 51 | 49 | 0.16 | 2014 | U ARAB EMIRATES |
| 52 | 44 | 0 | 2014 | LEBANON |
| 53 | 40 | 0.07 | 2014 | NIGERIA |
| 54 | 37 | 0.02 | 2023 | TURKIYE |
| 55 | 36 | 0 | 2014 | BULGARIA |
| 56 | 35 | 0 | 2014 | JORDAN |
| 57 | 34 | 0.35 | 2014 | INDONESIA |
| 58 | 34 | 0.05 | 2015 | VIETNAM |
| 59 | 32 | 0.06 | 2015 | QATAR |
| 60 | 30 | 0.7 | 2014 | UKRAINE |
| 61 | 29 | 0.07 | 2014 | TUNISIA |
| 62 | 29 | 0.05 | 2014 | SLOVAKIA |
| 63 | 28 | 0.07 | 2014 | SLOVENIA |
| 64 | 28 | 0.43 | 2015 | PHILIPPINES |
| 65 | 25 | 0 | 2017 | ETHIOPIA |
| 66 | 25 | 0.05 | 2014 | CYPRUS |
| 67 | 23 | 0.28 | 2014 | LITHUANIA |
| 68 | 23 | 0.05 | 2014 | BANGLADESH |
| 69 | 22 | 0.07 | 2014 | PERU |
| 70 | 19 | 0 | 2015 | NEPAL |
| 71 | 19 | 0 | 2015 | KENYA |
| 72 | 18 | 0.84 | 2014 | ECUADOR |
| 73 | 17 | 0 | 2014 | IRAQ |
| 74 | 16 | 0.09 | 2014 | ALGERIA |
| 75 | 16 | 0.07 | 2014 | ICELAND |
| 76 | 15 | 0.14 | 2015 | KUWAIT |
| 77 | 15 | 0 | 2018 | TANZANIA |
| 78 | 12 | 0.1 | 2016 | SUDAN |
| 79 | 11 | 0 | 2017 | GHANA |
| 80 | 11 | 0.38 | 2015 | LATVIA |
| 81 | 11 | 0.03 | 2015 | VENEZUELA |
| 82 | 11 | 0.02 | 2014 | MONTENEGRO |
| 83 | 10 | 0 | 2014 | NORTH MACEDONIA |
| 84 | 10 | 0.13 | 2014 | MOROCCO |
| 85 | 10 | 0.17 | 2015 | BAHRAIN |
| 86 | 10 | 0.03 | 2014 | OMAN |
| 87 | 10 | 0 | 2020 | CAMEROON |
| 88 | 9 | 0 | 2017 | LUXEMBOURG |
| 89 | 9 | 0.05 | 2017 | ZAMBIA |
| 90 | 9 | 0.13 | 2014 | URUGUAY |
| 91 | 9 | 0 | 2014 | ESTONIA |
| 92 | 8 | 0.13 | 2015 | CUBA |
| 93 | 8 | 0.07 | 2015 | SRI LANKA |
| 94 | 8 | 0 | 2017 | DEM REP CONGO |
| 95 | 8 | 0.17 | 2017 | GUATEMALA |
| 96 | 8 | 0.24 | 2019 | UGANDA |
| 97 | 7 | 0 | 2014 | BELARUS |
| 98 | 7 | 0 | 2014 | GEORGIA |
| 99 | 7 | 0.26 | 2015 | BOSNIA & HERCEG |
| 100 | 6 | 0 | 2017 | YEMEN |
| 101 | 6 | 0 | 2016 | PALESTINE |
| 102 | 5 | 0 | 2015 | KOSOVO |
| 103 | 5 | 0 | 2015 | ALBANIA |
| 104 | 5 | 0.09 | 2019 | MAURITIUS |
| 105 | 5 | 0.05 | 2014 | LIBYA |
| 106 | 5 | 0.07 | 2014 | SYRIA |
| 107 | 5 | 0.05 | 2017 | GRENADA |
| 108 | 5 | 0.04 | 2018 | BOTSWANA |
| 109 | 5 | 0 | 2016 | JAMAICA |
| 110 | 5 | 0 | 2016 | MONGOLIA |
| 111 | 5 | 0 | 2019 | MALTA |
| 112 | 4 | 0 | 2017 | MALAWI |
| 113 | 4 | 0.15 | 2020 | MOZAMBIQUE |
| 114 | 4 | 0.28 | 2021 | AFGHANISTAN |
| 115 | 4 | 0 | 2016 | KAZAKHSTAN |
| 116 | 3 | 0 | 2021 | BENIN |
| 117 | 3 | 0.01 | 2017 | ZIMBABWE |
| 118 | 3 | 0.02 | 2015 | LIECHTENSTEIN |
| 119 | 3 | 0.01 | 2014 | ARMENIA |
| 120 | 3 | 0.13 | 2018 | DOMINICAN REP |
| 121 | 3 | 0 | 2019 | NAMIBIA |
| 122 | 3 | 0.46 | 2018 | PARAGUAY |
| 123 | 2 | 0 | 2023 | TIMOR-LESTE |
| 124 | 2 | 0 | 2017 | MADAGASCAR |
| 125 | 2 | 0 | 2018 | BARBADOS |
| 126 | 2 | 0 | 2019 | TRINIDAD TOBAGO |
| 127 | 2 | 0 | 2021 | BURKINA FASO |
| 128 | 2 | 0 | 2015 | REP CONGO |
| 129 | 2 | 0 | 2016 | MACEDONIA |
| 130 | 2 | 0.19 | 2023 | BHUTAN |
| 131 | 2 | 0.13 | 2018 | EL SALVADOR |
| 132 | 2 | 0.02 | 2022 | RWANDA |
| 133 | 1 | 0 | 2018 | MYANMAR |
| 134 | 1 | 0 | 2024 | CAMBODIA |
| 135 | 1 | 0 | 2022 | COTE IVOIRE |
| 136 | 1 | 0 | 2021 | MOLDOVA |
| 137 | 1 | 0 | 2021 | TAJIKISTAN |
| 138 | 1 | 0 | 2022 | SENEGAL |
| 139 | 1 | 0 | 2020 | VATICAN |
| 140 | 1 | 0 | 2019 | GUYANA |
| 141 | 1 | 0 | 2023 | ANGOLA |
| 142 | 1 | 0 | 2015 | GABON |
| 143 | 1 | 0 | 2023 | SAN MARINO |
| 144 | 1 | 0 | 2017 | GAMBIA |
| 145 | 1 | 0 | 2017 | SURINAME |
| 146 | 1 | 0 | 2024 | FAROE ISLANDS |
| 147 | 1 | 0 | 2023 | PANAMA |
| 148 | 1 | 0 | 2017 | COSTA RICA |
| 149 | 1 | 0 | 2022 | UZBEKISTAN |
| 150 | 1 | 0 | 2019 | MONACO |
| 151 | 1 | 0 | 2020 | HAITI |
