# Supplementary material for: Global research trends and hotspots in aspirin studies (2014–2024): a bibliometric perspective
Source: Front Pharmacol. 2025 May 16;16:1513318. doi: 10.3389/fphar.2025.1513318 (PMC12123089; doi:10.3389/fphar.2025.1513318)
Supplement: Supplementary file 2 [file Table2.docx]

**S2. High frequency keywords**

| **Rank** | **Count** | **Centrality** | **Year** | **Keywords** |
| --- | --- | --- | --- | --- |
| 1 | 5539 | 0.13 | 2014 | aspirin |
| 2 | 2995 | 0.01 | 2014 | risk |
| 3 | 1916 | 0.01 | 2014 | prevention |
| 4 | 1892 | 0.22 | 2014 | clopidogrel |
| 5 | 1793 | 0.04 | 2014 | low dose aspirin |
| 6 | 1503 | 0.64 | 2014 | management |
| 7 | 1498 | 0 | 2014 | therapy |
| 8 | 1279 | 0.03 | 2014 | antiplatelet therapy |
| 9 | 1261 | 0.23 | 2014 | myocardial infarction |
| 10 | 1158 | 0 | 2014 | disease |
| 11 | 1109 | 0.1 | 2014 | outcome |
| 12 | 1056 | 0.06 | 2014 | mortality |
| 13 | 1039 | 1.15 | 2014 | percutaneous coronary intervention |
| 14 | 1024 | 1.13 | 2014 | atrial fibrillation |
| 15 | 1020 | 0 | 2014 | metaanalysis |
| 16 | 1014 | 0 | 2014 | risk factors |
| 17 | 967 | 0.7 | 2014 | nonsteroidal antiinflammatory drugs |
| 18 | 960 | 0 | 2014 | association |
| 19 | 894 | 0.1 | 2014 | dual antiplatelet therapy |
| 20 | 822 | 0.57 | 2014 | expression |
| 21 | 817 | 0.24 | 2014 | inhibition |
| 22 | 802 | 0 | 2014 | cardiovascular disease |
| 23 | 798 | 0.03 | 2014 | double blind |
| 24 | 760 | 0.2 | 2014 | warfarin |
| 25 | 720 | 0.03 | 2014 | acetylsalicylic acid |
| 26 | 680 | 0.7 | 2014 | colorectal cancer |
| 27 | 659 | 0.25 | 2014 | activation |
| 28 | 652 | 0.03 | 2014 | events |
| 29 | 641 | 0.11 | 2014 | inflammation |
| 30 | 630 | 0.03 | 2014 | coronary artery disease |
| 31 | 623 | 0.01 | 2014 | stroke |
| 32 | 604 | 0.54 | 2014 | guidelines |
| 33 | 580 | 0.21 | 2014 | secondary prevention |
| 34 | 578 | 0.03 | 2014 | prevalence |
| 35 | 571 | 0 | 2014 | efficacy |
| 36 | 567 | 0.06 | 2014 | women |
| 37 | 566 | 0.23 | 2014 | ischemic stroke |
| 38 | 544 | 0.01 | 2014 | acute coronary syndrome |
| 39 | 543 | 0 | 2014 | acute myocardial infarction |
| 40 | 534 | 0.01 | 2014 | impact |
| 41 | 523 | 0.2 | 2014 | primary prevention |
| 42 | 514 | 0 | 2014 | diagnosis |
| 43 | 489 | 0 | 2014 | antithrombotic therapy |
| 44 | 486 | 0 | 2014 | trial |
| 45 | 481 | 0.98 | 2014 | cardiovascular events |
| 46 | 470 | 0.03 | 2014 | thrombosis |
| 47 | 457 | 0.01 | 2014 | safety |
| 48 | 452 | 0 | 2014 | complications |
| 49 | 446 | 0.01 | 2014 | venous thromboembolism |
| 50 | 433 | 0 | 2014 | ticagrelor |
| 51 | 401 | 0 | 2014 | intervention |
| 52 | 401 | 0 | 2014 | randomized trial |
| 53 | 394 | 0 | 2014 | cells |
| 54 | 387 | 0.62 | 2014 | survival |
| 55 | 386 | 0.04 | 2014 | oxidative stress |
| 56 | 384 | 0.11 | 2014 | resistance |
| 57 | 379 | 0.04 | 2014 | in vitro |
| 58 | 373 | 0.04 | 2014 | cancer |
| 59 | 351 | 0.3 | 2014 | artery disease |
| 60 | 349 | 0 | 2014 | mechanisms |
| 61 | 334 | 0.16 | 2014 | platelet reactivity |
| 62 | 328 | 0 | 2014 | aspirin use |
| 63 | 326 | 0.01 | 2014 | surgery |
| 64 | 322 | 0 | 2014 | platelet aggregation |
| 65 | 320 | 0.06 | 2014 | molecular weight heparin |
| 66 | 311 | 0.63 | 2014 | breast cancer |
| 67 | 311 | 0.03 | 2014 | prasugrel |
| 68 | 300 | 0.32 | 2014 | rivaroxaban |
| 69 | 292 | 0.01 | 2014 | acid |
| 70 | 279 | 0.49 | 2014 | transient ischemic attack |
| 71 | 272 | 0.04 | 2014 | aggregation |
| 72 | 272 | 0.03 | 2014 | aspirin resistance |
| 73 | 269 | 0.21 | 2014 | blood pressure |
| 74 | 267 | 0 | 2014 | follow up |
| 75 | 267 | 0 | 2014 | population |
| 76 | 265 | 0.03 | 2014 | nitric oxide |
| 77 | 264 | 0.03 | 2014 | growth |
| 78 | 264 | 0 | 2014 | oral anticoagulants |
| 79 | 262 | 0.03 | 2014 | acute coronary syndrm |
| 80 | 257 | 0 | 2014 | platelet function |
| 81 | 251 | 0.41 | 2014 | nf kappa b |
| 82 | 250 | 0.04 | 2014 | diabetes mellitus |
| 83 | 249 | 0 | 2014 | pregnancy |
| 84 | 245 | 0.18 | 2014 | apoptosis |
| 85 | 245 | 0.01 | 2014 | acute ischemic stroke |
| 86 | 241 | 0.13 | 2014 | preeclampsia |
| 87 | 228 | 0.04 | 2015 | high risk |
| 88 | 227 | 0 | 2014 | reactivity |
| 89 | 225 | 0.04 | 2014 | anticoagulation |
| 90 | 219 | 0 | 2014 | task force |
| 91 | 213 | 0 | 2014 | clinical outcm |
| 92 | 210 | 0.06 | 2014 | chronic rhinosinusitis |
| 93 | 209 | 0 | 2014 | randomized controlled trial |
| 94 | 206 | 0.74 | 2014 | proton pump inhibitors |
| 95 | 204 | 0 | 2014 | platelet inhibition |
| 96 | 203 | 0.04 | 2014 | drugs |
| 97 | 199 | 0 | 2015 | health |
| 98 | 198 | 0.01 | 2014 | implantation |
| 99 | 194 | 0.01 | 2014 | validation |
| 100 | 193 | 0.1 | 2014 | cyclooxygenase 2 |
| 101 | 192 | 0 | 2014 | predictors |
| 102 | 190 | 0 | 2015 | salicylic acid |
| 103 | 187 | 0.55 | 2014 | dabigatran |
| 104 | 186 | 0 | 2014 | platelet activation |
| 105 | 185 | 0.33 | 2018 | focused update |
| 106 | 185 | 0 | 2014 | mechanism |
| 107 | 183 | 0 | 2014 | multicenter |
| 108 | 182 | 0 | 2014 | atherosclerosis |
| 109 | 182 | 0 | 2014 | deep vein thrombosis |
| 110 | 181 | 0.06 | 2015 | epidemiology |
| 111 | 172 | 0.01 | 2014 | colon cancer |
| 112 | 172 | 0 | 2014 | american college |
| 113 | 171 | 0 | 2014 | coronary heart disease |
| 114 | 161 | 0.01 | 2014 | model |
| 115 | 161 | 0 | 2014 | pharmacokinetics |
| 116 | 157 | 0.88 | 2018 | open label |
| 117 | 154 | 0 | 2014 | heart failure |
| 118 | 152 | 0.01 | 2014 | prediction |
| 119 | 144 | 0.52 | 2018 | minor stroke |
| 120 | 144 | 0.06 | 2014 | elevation myocardial infarction |
| 121 | 143 | 0 | 2015 | oral anticoagulation |
| 122 | 141 | 0.01 | 2014 | chronic kidney disease |
| 123 | 134 | 0.13 | 2014 | stroke prevention |
| 124 | 130 | 0.03 | 2014 | heart disease |
| 125 | 130 | 0.01 | 2015 | antiplatelet |
| 126 | 128 | 0.03 | 2015 | design |
| 127 | 125 | 0.14 | 2015 | hypertension |
| 128 | 125 | 0.08 | 2014 | united states |
| 129 | 124 | 0.06 | 2014 | inhibitors |
| 130 | 122 | 0 | 2014 | receptor |
| 131 | 117 | 0.03 | 2018 | kawasaki disease |
| 132 | 113 | 0 | 2015 | identification |
| 133 | 109 | 0.05 | 2014 | pulmonary embolism |
| 134 | 108 | 0 | 2014 | children |
| 135 | 106 | 0.01 | 2014 | nasal polyps |
| 136 | 105 | 0.55 | 2016 | metastasis |
| 137 | 98 | 0 | 2014 | cohort |
| 138 | 98 | 0 | 2014 | stent thrombosis |
| 139 | 96 | 0 | 2014 | antiplatelet agents |
| 140 | 95 | 0 | 2014 | c reactive protein |
| 141 | 91 | 0.04 | 2014 | antiphospholipid syndrome |
| 142 | 90 | 0 | 2014 | risk factor |
| 143 | 89 | 0.03 | 2014 | association task force |
| 144 | 89 | 0 | 2014 | st segment elevation |
| 145 | 88 | 0 | 2014 | injury |
| 146 | 84 | 0.06 | 2014 | prophylaxis |
| 147 | 75 | 0 | 2014 | care |
| 148 | 71 | 0.1 | 2019 | replacement |
| 149 | 71 | 0 | 2016 | aspirin-exacerbated respiratory disease |
| 150 | 71 | 0 | 2018 | asthma |
| 151 | 69 | 0.08 | 2015 | adults |
| 152 | 69 | 0 | 2019 | classification |
| 153 | 68 | 0 | 2014 | responsiveness |
| 154 | 67 | 0 | 2014 | platelet aggregation inhibitors |
| 155 | 65 | 0 | 2020 | duration |
| 156 | 60 | 0.04 | 2022 | peripheral artery disease |
| 157 | 60 | 0 | 2020 | placebo |
| 158 | 58 | 0 | 2016 | eluting stent implantation |
| 159 | 55 | 0.03 | 2020 | hypertensive disorders |
| 160 | 53 | 0 | 2017 | systemic lupus erythematosus |
| 161 | 52 | 0.09 | 2014 | pathogenesis |
| 162 | 52 | 0 | 2016 | exacerbated respiratory disease |
| 163 | 51 | 0 | 2014 | controlled trial |
| 164 | 51 | 0 | 2014 | progression |
| 165 | 50 | 0.01 | 2015 | essential thrombocythemia |
| 166 | 50 | 0 | 2014 | dysfunction |
| 167 | 49 | 0.03 | 2014 | uterine artery doppler |
| 168 | 49 | 0 | 2014 | in vivo |
| 169 | 48 | 0.01 | 2017 | european society |
| 170 | 48 | 0 | 2014 | acetaminophen |
| 171 | 47 | 0.01 | 2014 | chemoprevention |
| 172 | 47 | 0 | 2015 | society |
| 173 | 45 | 0.92 | 2018 | triple therapy |
| 174 | 45 | 0.19 | 2019 | thromboprophylaxis |
| 175 | 45 | 0 | 2014 | celecoxib |
| 176 | 44 | 0 | 2019 | delivery |
| 177 | 41 | 0 | 2015 | hemorrhage |
| 178 | 38 | 0 | 2019 | recurrence |
| 179 | 37 | 0 | 2021 | cardiovascular diseases |
| 180 | 37 | 0 | 2022 | hepatocellular carcinoma |
| 181 | 36 | 0.01 | 2014 | peripheral arterial disease |
| 182 | 36 | 0 | 2016 | clinical trials |
| 183 | 36 | 0 | 2014 | clopidogrel resistance |
| 184 | 36 | 0 | 2015 | death |
| 185 | 36 | 0 | 2019 | polymorphisms |
| 186 | 36 | 0 | 2014 | protein |
| 187 | 36 | 0 | 2015 | quality of life |
| 188 | 35 | 0.1 | 2017 | resolution |
| 189 | 35 | 0.04 | 2020 | pci |
| 190 | 35 | 0 | 2021 | coronary |
| 191 | 34 | 0.11 | 2015 | intracranial hemorrhage |
| 192 | 34 | 0.03 | 2019 | release |
| 193 | 33 | 0 | 2014 | elderly patients |
| 194 | 33 | 0 | 2017 | trends |
| 195 | 32 | 0.01 | 2014 | gene expression |
| 196 | 32 | 0 | 2014 | adherence |
| 197 | 32 | 0 | 2015 | apixaban |
| 198 | 32 | 0 | 2015 | arachidonic acid |
| 199 | 32 | 0 | 2014 | body mass index |
| 200 | 32 | 0 | 2019 | collaborative metaanalysis |
| 201 | 32 | 0 | 2015 | noncardiac surgery |
| 202 | 32 | 0 | 2014 | predicting stroke |
| 203 | 31 | 0.04 | 2023 | reducing events |
| 204 | 31 | 0.01 | 2014 | cost effectiveness |
| 205 | 30 | 0.03 | 2014 | damage |
| 206 | 28 | 0.73 | 2014 | helicobacter pylori infection |
| 207 | 21 | 0.01 | 2015 | platelet function tests |
| 208 | 20 | 0.04 | 2023 | preterm |
| 209 | 20 | 0 | 2023 | case report |
| 210 | 20 | 0 | 2020 | metabolism |
| 211 | 19 | 0.03 | 2023 | score |
| 212 | 19 | 0 | 2022 | health care professionals |
| 213 | 19 | 0 | 2022 | platelets |
| 214 | 18 | 0 | 2023 | gut microbiota |
| 215 | 18 | 0 | 2021 | proliferation |
| 216 | 18 | 0 | 2014 | prostate cancer |
| 217 | 18 | 0 | 2023 | system |
| 218 | 17 | 0.07 | 2021 | enoxaparin |
| 219 | 17 | 0.01 | 2019 | nanoparticles |
| 220 | 17 | 0 | 2020 | alzheimers disease |
| 221 | 17 | 0 | 2021 | direct oral anticoagulants |
| 222 | 17 | 0 | 2015 | helicobacter pylori |
| 223 | 17 | 0 | 2021 | infection |
| 224 | 17 | 0 | 2021 | mellitus |
| 225 | 17 | 0 | 2015 | nitric oxide synthase |
| 226 | 17 | 0 | 2015 | peptic ulcer |
| 227 | 17 | 0 | 2015 | rheumatoid arthritis |
| 228 | 17 | 0 | 2021 | trials |
| 229 | 17 | 0 | 2014 | unfractionated heparin |
| 230 | 17 | 0 | 2016 | unstable angina |
| 231 | 16 | 0.01 | 2022 | attack |
| 232 | 16 | 0.01 | 2015 | derivatives |
| 233 | 16 | 0 | 2015 | antiplatelet drugs |
| 234 | 16 | 0 | 2015 | cardiovascular risk |
| 235 | 16 | 0 | 2014 | cyclooxygenase 2 expression |
| 236 | 16 | 0 | 2014 | drug eluting stents |
| 237 | 16 | 0 | 2022 | fetal growth restriction |
| 238 | 16 | 0 | 2022 | heparin |
| 239 | 16 | 0 | 2015 | low-dose aspirin |
| 240 | 16 | 0 | 2020 | obesity |
| 241 | 16 | 0 | 2017 | pain |
| 242 | 16 | 0 | 2016 | placebo controlled trial |
| 243 | 16 | 0 | 2024 | systematic review |
| 244 | 16 | 0 | 2016 | time |
| 245 | 16 | 0 | 2014 | update |
| 246 | 15 | 0 | 2015 | cardiac surgery |
| 247 | 15 | 0 | 2015 | dose clopidogrel |
| 248 | 15 | 0 | 2014 | mice |
| 249 | 15 | 0 | 2014 | non-steroidal anti-inflammatory drugs |
| 250 | 15 | 0 | 2019 | performance |
| 251 | 15 | 0 | 2015 | polymorphism |
| 252 | 15 | 0 | 2014 | responses |
| 253 | 14 | 0 | 2015 | agents |
| 254 | 14 | 0 | 2014 | infarction |
| 255 | 14 | 0 | 2014 | pathway |
| 256 | 14 | 0 | 2014 | plasma |
| 257 | 14 | 0 | 2015 | quality |
| 258 | 14 | 0 | 2015 | stent implantation |
| 259 | 13 | 0.52 | 2024 | alteplase |
| 260 | 13 | 0.09 | 2015 | age |
| 261 | 13 | 0 | 2014 | antibody |
| 262 | 13 | 0 | 2015 | bleeding complications |
| 263 | 13 | 0 | 2014 | combination |
